# The PP2A regulator IER5L supports prostate cancer progression

**DOI:** 10.1038/s41419-024-06907-z

**Published:** 2024-07-18

**Authors:** Jana R. Crespo, Natalia Martín-Martín, Saioa Garcia-Longarte, Jon Corres-Mendizabal, Onintza Carlevaris, Ianire Astobiza, Amaia Zabala-Letona, Marc Guiu, Mikel Azkargorta, Monika Gonzalez-Lopez, Nuria Macías-Cámara, Phuong Doan, Félix Elortza, Isabel Mendizabal, Jukka Westermarck, Roger R. Gomis, Amaia Ercilla, Arkaitz Carracedo

**Affiliations:** 1 Center for Cooperative Research in Biosciences (CIC bioGUNE), Basque Research and Technology Alliance (BRTA), Bizkaia Technology Park, Derio, Spain; 2 Traslational prostate cancer Research lab, CIC bioGUNE-Basurto, Biobizkaia Health Research Institute, Bizkaia, Spain; 3 Centro de Investigación Biomédica En Red de Cáncer (CIBERONC), Madrid, Spain; 4 Cancer Science Program, Institute for Research in Biomedicine (IRB Barcelona), The Barcelona Institute of Science and Technology, Barcelona, Spain; 5 Proteomics Platform, CIC bioGUNE, Basque Research and Technology Alliance (BRTA), Gipuzkoa, Spain; 6 CIBERehd, Bizkaia Science and Technology Park, Derio, Spain; 7 Turku Bioscience Centre, University of Turku and Åbo Akademi University, Turku, Finland; 8 IKERBASQUE, Basque Foundation for Science, Bilbao, Spain; 9 Institute of Biomedicine and InFLAMES Research Flagship, University of Turku, Turku, Finland; 10 School of Medicine, Universitat de Barcelona, Barcelona, Spain; 11 ICREA, Institució Catalana de Recerca i Estudis Avançats, Barcelona, Spain; 12 Biochemistry and Molecular Biology Department, University of the Basque Country (UPV/EHU), Bilbao, Spain

**Keywords:** Cell signalling, Metastasis

## Abstract

Prostate cancer exhibits high prevalence and accounts for a high number of cancer-related deaths. The discovery and characterization of molecular determinants of aggressive prostate cancer represents an active area of research. The Immediate Early Response (IER) family of genes, which regulate Protein Phosphatase 2A (PP2A) activity, has emerged among the factors that influence cancer biology. Here, we show that the less studied member of this family, Immediate Early Response 5 like (IER5L), is upregulated in aggressive prostate cancer. Interestingly, the upregulation of *IER5L* expression exhibits a robust association with metastatic disease in prostate and is recapitulated in other cancer types. In line with this observation, *IER5L* silencing reduces foci formation, migration and invasion ability in a variety of human and murine prostate cancer cell lines. In vivo, using zebrafish and immunocompromised mouse models, we demonstrate that *IER5L*-silencing reduces prostate cancer tumor growth, dissemination, and metastasis. Mechanistically, we characterize the transcriptomic and proteomic landscapes of *IER5L*-silenced cells. This approach allowed us to identify DNA replication and monomeric G protein regulators as downstream programs of IER5L through a pathway that is consistent with the regulation of PP2A. In sum, we report the alteration of IER5L in prostate cancer and beyond and provide biological and molecular evidence of its contribution to tumor aggressiveness.

## Introduction

Nearly 10 million people are estimated to die of cancer every year. The prognosis for an individual with cancer is highly variable and dependent on tumor type, grade and stage at primary diagnosis [1]. Alterations in the genome, gene expression levels, and protein structure or function can be used as molecular markers to predict the outcome of patients before the advent of recurrence [2]. The identification of those prognostic features is essential to anticipate the disease trajectory of a patient and overcome the high mortality rates due to the emergence of metastasis. From the different tumor types, prostate cancer (PCa) is the second most common cancer in men, causing over 350,000 deaths worldwide every year [3]. Metastasis is the main cause of morbidity and mortality associated with this disease. Although life expectancy for men with localized tumors has substantially increased thanks to early detection and the increased efficacy of first-line therapies, the identification of the patients at high risk of recurrence is still a challenge since specific molecular alterations that distinguish aggressive from indolent PCa are difficult to establish [3].

Immediate-early response (IER) family genes encode a variety of factors that are involved in diverse cellular functions including differentiation, metabolism and proliferation [4]. IER family genes are cell cycle regulated and their protein products are typically unstable and rapidly targeted for proteasomal degradation. However, many tumor types present abnormally high expression of IER genes [4]. Immediate Early Response 2 (IER2), Immediate Early Response 5 (IER5) and Immediate Early Response 5 like (IER5L) are three IER family members that share homology at their N-terminal region. All three proteins interact with the B55 regulatory subunit of Protein phosphatase 2A (PP2A) through their N-terminal region [5]. They also bind to the target proteins of PP2A and assist PP2A-mediated dephosphorylation [5–8]. Interestingly, both IER2 and IER5 reportedly contribute to tumor progression. High IER5 levels induce abnormal Heat Shock Transcription Factor 1 (HSF1) activation to sustain proliferation under stress conditions [9]. Likewise, IER2 has been proposed to participate in tumorigenic processes such as cell motility and adhesion, angiogenesis, invasion and metastasis in several cancer types, including colorectal and hepatocellular carcinoma [10–13]. Unlike IER2 and IER5, the role of IER5L in cancer aggressiveness has been poorly studied.

In this study, we exploit bioinformatics analysis of public transcriptomics datasets to find a consistent and unique association of *IER5L* expression among the IER family genes with tumor pathogenesis, progression and metastasis. Our results show that *IER5L* is a prognostic gene whose expression is upregulated across different cancer types, with a remarkable overexpression in metastatic PCa. Taking advantage of cellular systems, we show that IER5L sustains the proliferation under stress, migration and invasion capacity of PCa cells, and that its depletion compromises tumor growth and dissemination in vivo using zebrafish and murine models. Mechanistically, *IER5L* silencing elicits molecular alterations that are consistent with the inhibition of PP2A and the repression of regulators of monomeric G proteins. Collectively, our work uncovers IER5L as a novel IER family gene that regulates PCa progression.

## Results

### IER5L is upregulated in cancer

**Fig. 1:**
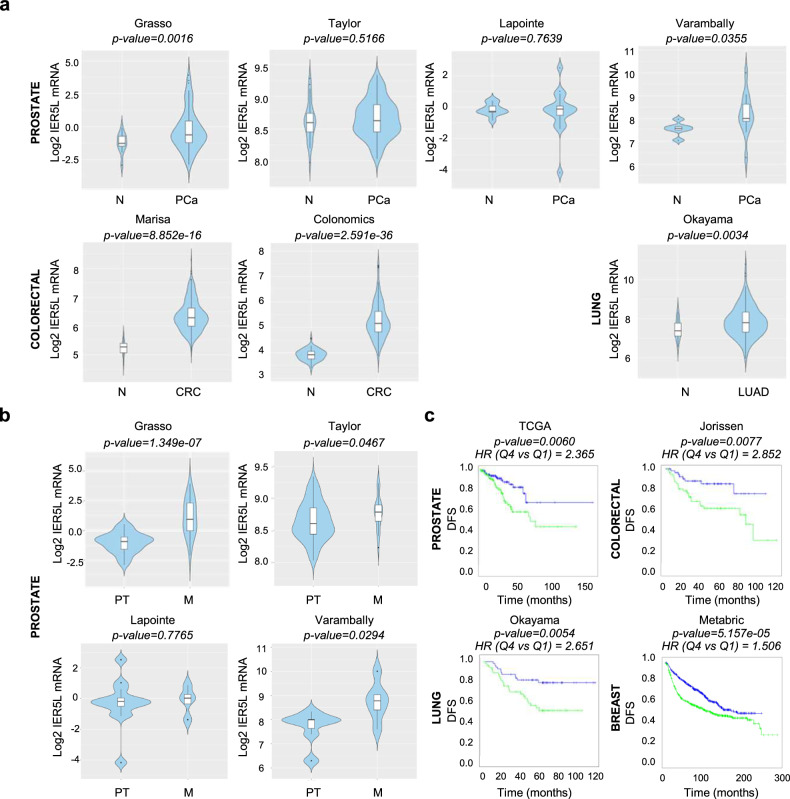
IER5L is upregulated in metastatic prostate cancer. **a** Violin plots depicting the Log2 expression of *IER5L* in non-tumoral (N), prostate cancer (PCa), lung adenocarcinoma (LUAD) and colorectal cancer (CRC) specimens in the indicated dataset. *p*-value derives from a Student’s *t*-test analysis between the indicated groups. **b** Violin plots showing the Log2 expression of *IER5L* mRNA in primary tumor (PT) and metastatic (M) PCa specimens. p-value derives from a Student’s *t*-test analysis between the indicated groups. **c** Kaplan-Meyer curves showing the association of *IER5L* mRNA expression to disease-free survival (DFS) in the indicated datasets. Quartile 1 (blue) and quartile 4 (green) are represented. A log-rank test *p*-value and the hazard ratio (HR) between two groups calculated by Cox proportional hazard model regression are provided above each graph.

To analyze the association of IER family members (IER2, IER5 and IER5L) with tumor pathogenesis and progression, we interrogated public transcriptomics datasets containing gene expression data from different tumor types (Supplementary Table S1) using Cancertool [14]. *IER2* and *IER5* expression was altered in some of the prostate, colorectal and lung datasets analyzed, but did not show a directional consistency across tumor types (Supplementary Fig. S1). By contrast, *IER5L* was consistently upregulated in colorectal (2/2 datasets), lung (1/1 datasets) and prostate (2/4 datasets) tumor specimens when compared to non-cancerous tissue (Fig. 1a), consistent with recent reports [15, 16]. To further explore whether the increase in IER5L levels was a common feature of different tumor types, we took advantage of TIMER web interface [17], which allowed us to explore the cancer-associated alterations in the TCGA cohorts. *IER5L* levels consistently increased across cancer types (with a similar non-significant trend in PCa), while *IER2* expression was largely reduced, and *IER5* exhibited inconsistent alterations (Supplementary Fig. S2).

Unlike other datasets, the PCa datasets included in Cancertool contain the gene expression information of metastatic samples. To decipher whether the changes in the expression of IER family genes were emphasized in metastatic specimens, we compared the gene expression of 56 metastatic samples and 200 primary tumors. Remarkably, *IER5L* was increased in metastatic specimens in 3 out of 4 of the interrogated prostate datasets (Fig. 1b), whereas *IER2* expression was predominantly reduced, and *IER5* exhibited no consistent pattern (Supplementary Fig. S3).

Next, taking advantage of the clinical follow-up information in various datasets, we interrogated the prognostic potential of the IER gene family by means of disease-free survival. Interestingly, IER5L exhibited the most prominent prognostic capacity when upregulated among the three members of the family (Fig. 1c, Supplementary Fig. S4). Taking into consideration the high consistency of IER5L upregulation in the various pathogenic scenarios analyzed, and the limited information about its role in cancer, we decided to study the biological and molecular function of IER5L using PCa as a tumor type where its alteration could be relevant.

### Silencing of *IER5L* compromises the foci formation, migration and invasion ability of PCa cells

**Fig. 2:**
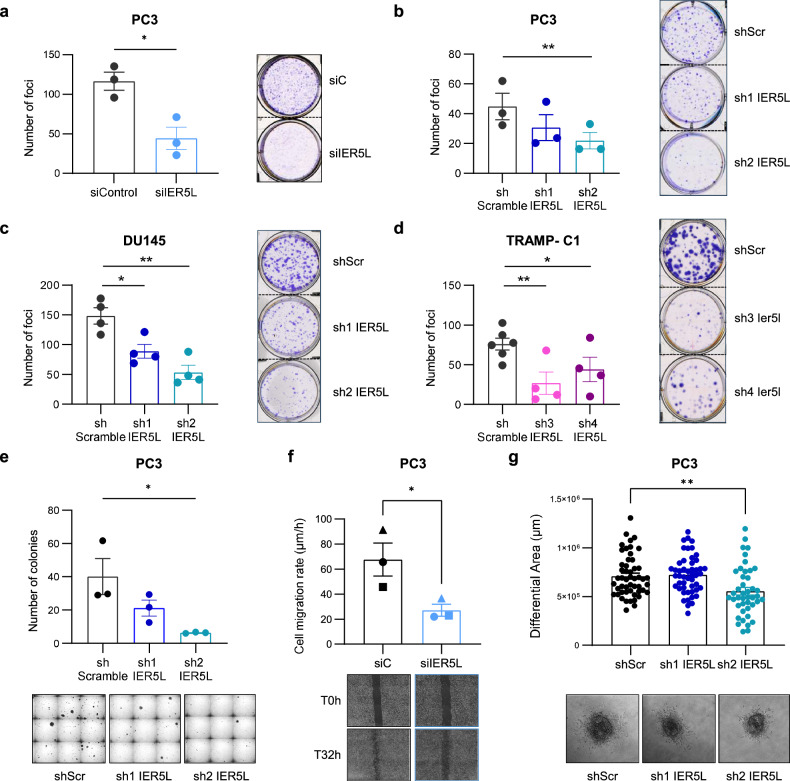
IER5L depletion reduces growth under stress, migration and invasion of prostate cancer cells. **a** Foci-formation analysis of PC3 cells transfected with the indicated siRNA. The number of foci is shown (left panels). Representative images are shown (right panels). A *t*-test was applied for statistical analysis (*n* = 3). siC: non-target siRNA. **b**–**d** Analysis of foci formation upon IER5L depletion with the indicated shRNAs. The number of foci is shown (left panels). Representative images are shown (right panels). A paired *t*-test was applied for statistical analysis (**b** *n* = 3; **c**, **d** *n* = 4). shScr: shScramble. **e** Analysis of anchorage-independent growth of PC3 cells transduced with the indicated shRNAs. The number of colonies is shown (left panel). Representative images are shown (right panel). A paired *t*-test was applied for statistical analysis (*n* = 3). **f** Cell migration rate of PC3 cells transfected with the indicated pool of siRNAs. The different biological replicates are indicated with unique dot shapes (top panel). Representative images of the scratch at time (T) 0 and 32 hours (h) are shown (bottom panel). Two days after siRNA transfection were defined as the initial timepoint for the assay. A two-tailed paired *t*-test was applied for statistical analysis (*n* = 3). **g** Quantification of the invasive growth of PC3 cells embedded in collagen. The diameter of the spheroids was measured at 0 and 72-h and the differential area was calculated (top panel). Representative images of the spheroids at final timepoint are shown (bottom panel). A two-tailed unpaired *t*-test was applied for statistical analysis (*n* = 4).

To test the role of IER5L in PCa aggressiveness, we analyzed the biological consequences of *IER5L* silencing in vitro. A decrease in *IER5L* mRNA levels by either small interference RNA (siRNA) transfection or short hairpin (shRNA) transduction did not compromise the overall cell growth of PC3 cells (Supplementary Fig. S5a, b). In contrast, when cells with decreased IER5L levels were forced to grow individualized, they showed a lower capability to form colonies in foci formation assays (Fig. 2a, b). These results were replicated in two additional human PCa cell lines (DU145 and 22RV1) and a murine PCa cell line, TRAMP-C1 (Fig. 2c, d, Supplementary Fig. S5c-f). Likewise, *IER5L* silencing also impaired the ability of human and murine PCa cells to grow in an anchorage-independent manner (Fig. 2e, Supplementary Fig. S5g). Of note, the silencing efficacy of the two human *IER5L*-targetting shRNAs was associated with the proportional tumor suppressive phenotype (Fig. 2, Supplementary Fig. S5). Moreover, consistent with the essential role of IER5L in sustaining cell proliferation, its silencing was progressively lost with passages (Supplementary Fig. S5h), suggestive of the negative selection of silenced cells in vitro and preventing us from generating CRISPR/Cas9-based *IER5L* knockout cells.

Besides the ability to grow under stress conditions, metastatic cells commonly acquire migration and invasion abilities that contribute to their metastatic potential. Thus, we ascertained the contribution of IER5L to these processes in PC3 cells. On one hand, IER5L-targeting siRNA transfection in PCa cells decreased their migration rate in wound healing assays (Fig. 2f). On the other hand, *IER5L* silencing reduced the invasive capacity of PC3 cells in spheroid assays using a collagen matrix, which was significant in cells with the greatest *IER5L* silencing (Fig. 2g, Supplementary Fig. S5b). Altogether, these data suggest that IER5L sustains aggressiveness properties in PCa.

### IER5L contributes to tumor growth and metastasis in vivo

**Fig. 3:**
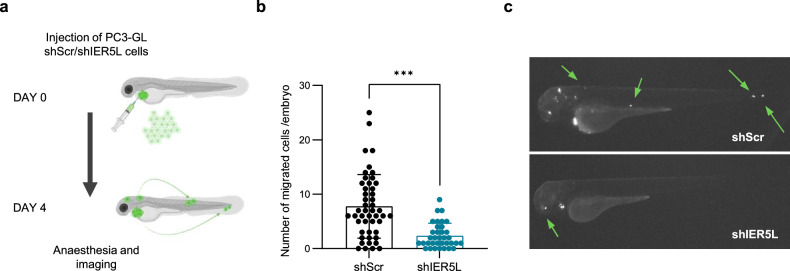
Prostate cancer cell dissemination is compromised upon IER5L depletion in zebrafish. **a** Representation of the experimental design of the in vivo cell dissemination assay in zebrafish. PC3 GFP-Luc (GL) cells transduced with shScramble (shScr) or sh2 IER5L (shIER5L) were injected into the pericardial cavity of zebrafish embryos. Cell dissemination was analyzed at 4 days post injection. The number of disseminated cells (**b**) and a representative image (**c**) are shown. A two-tailed Mann–Whitney test was applied for statistical analysis.

Our bioinformatics analysis revealed an association of *IER5L* upregulation to cancer progression (Fig. 1). In addition, in vitro assays showed a requirement of IER5L for cell growth under stress, migration and invasion. In turn, we designed in vivo strategies that enabled us to monitor all these different parameters. As a first approach, we exploited zebrafish model to evaluate the causal contribution of IER5L to PCa cell dissemination in vivo. The transparency of the young embryos, their lack of immune system and the rapid capacity to form primary tumor and metastases, makes zebrafish a suitable and attractive model for investigating tumor cell dissemination from the primary site [18]. PC3 cells stably expressing GFP-luciferase (GL) transduced with either shScramble or shIER5L were injected into the pericardial cavity of zebrafish embryos. The number of disseminated cells was analyzed at 4 days post injection (Fig. 3a). As shown in Fig. 3b, c, the silencing of *IER5L* significantly decreased the dissemination ability of PC3 cells in this model.

**Fig. 4:**
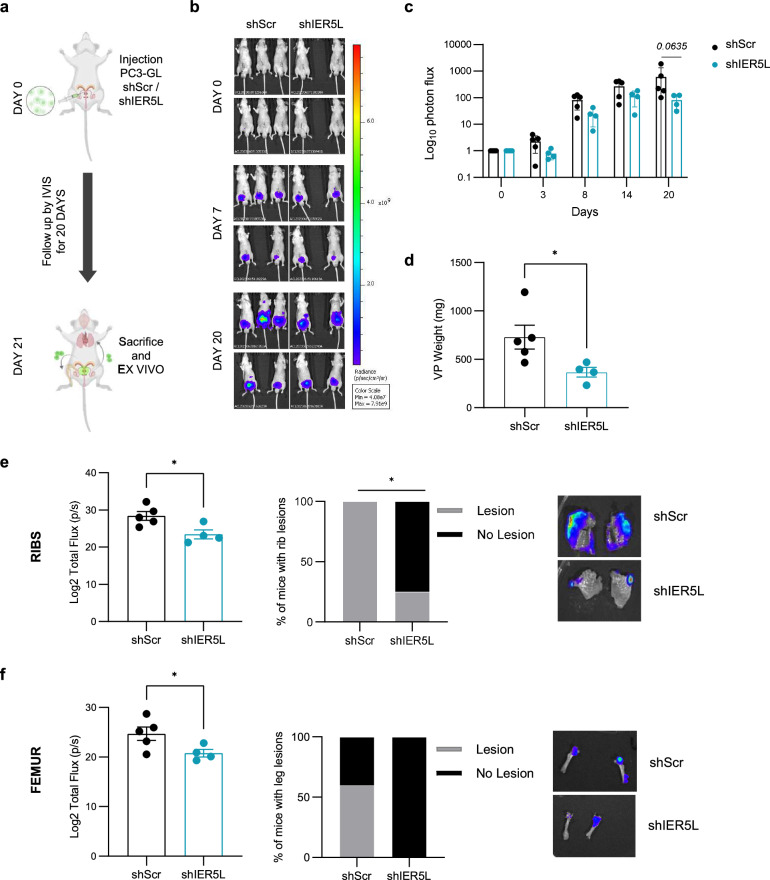
Silencing of *IER5L* counteracts cell growth and metastatic dissemination of prostate cancer cells in vivo. **a** Experimental design of the in vivo orthotopic assay. PC3 GFP-LUC (GL) cells transduced with shScramble (shScr) or sh2 IER5L (shIER5L) were injected into the ventral prostate lobe of nude mice and followed up for 20 days. IVIS relative flux data along the experimental process. Representative images (**b**) and the total photon flux normalized to time 0 (**c**) are represented. A multiple Mann-Whitney *U*-test was applied for statistical analysis. **d** Ex vivo tumor weight of the ventral lobes of prostates (VP) from the in vivo orthotopic assay. A two-tailed Mann-Whitney test was applied for statistical analysis. Ex vivo IVIS signal quantification of ribs (**e**) and femur (**f**) (left panels). Contingency analysis of metastatic lesions at those sites (middle panels) and a representative image (right panels) are shown. A one-tailed Mann–Whitney test and a Fisher exact t-test were used, respectively.

To further validate the causal contribution of IER5L to PCa cell growth and dissemination, we performed an in vivo orthotopic assay where PC3-GL cells were transduced with Scramble or IER5L-targeting shRNA (Fig. 4a). Luciferase-expressing cells were then injected into the ventral lobe of immune-deficient nude mice and tumor mass was monitored by in vivo imaging for 20 days. *IER5L*-silenced cells showed a lower luciferase signal throughout the experiment (Fig. 4b, c), which correlated with a reduced primary tumor weight at the experimental endpoint (Fig. 4d). Importantly, the analysis of luciferase signal in distal organs confirmed a lower metastatic burden in mice injected with *IER5L*-silenced PCa cells. This phenotype was more prominent in bones, including ribs and femur (Fig. 4e, f, Supplementary Fig. S6).

Altogether, these data underline the importance of IER5L in sustaining metastatic properties in PCa cells.

### *IER5L* silencing elicits molecular alterations consistent with PP2A inhibition

**Fig. 5:**
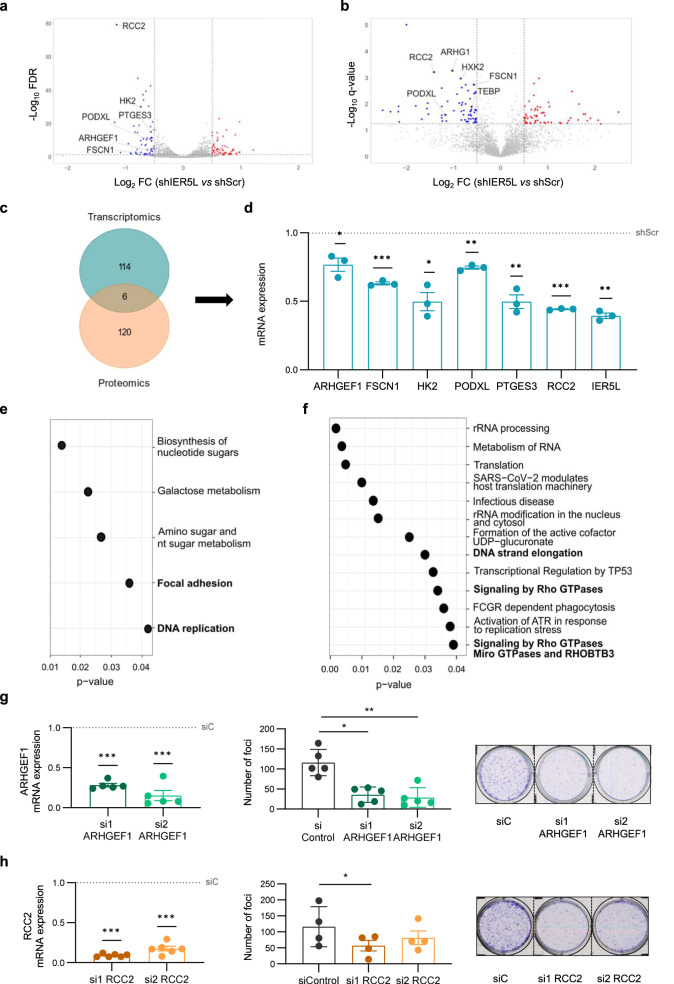
IER5L depletion targets DNA replication and monomeric G protein pathways. Volcano plot representation of the differentially expressed genes (DEGs) (**a**) and proteins (**b**) upon *IER5L* silencing by shScramble (shScr) or sh2 IER5L (shIER5L) transduction. The common targets between the RNAseq and proteomics’ analyses are highlighted. **c** Venn diagram summarizing the number of DEGs and the proteins affected by IER5L depletion in the RNAseq and proteomics experiments from (**a**) and (**b**). **d** Analysis of the expression of the indicated genes by qRT-PCR upon IER5L depletion by sh2 IER5L transduction in PC3 cells. The mRNA levels are normalized to *GAPDH* and shScr. The dotted line represents the normalized value of the shScr data. A one-sample *t*-test was applied for statistical analysis (*n* = 3). Functional enrichment analysis of the DEGs upon *IER5L* silencing by KEGG (**e**) and Reactome (**f**). Left panels: Analysis of ARHGEF1 (**g**) and RCC2 (**h**) mRNA expression by qRT-PCR. The levels are normalized to *GAPDH* and non-target (siC) condition. The dotted line represents the normalized value of the siC data. A one-sample *t*-test was applied for statistical analysis (*n* = 5 and *n* = 6 respectively). Middle panels: Analysis of foci formation upon ARHGEF1 (**g**) and RCC2 (**h**) depletion. The number of foci is shown. A paired *t*-test was applied for statistical analysis (*n* = 5 and *n* = 4 respectively). Right panels: Representative images of the foci experiments are shown.

To gain insight into the molecular mechanism by which IER5L contributes to the acquisition of aggressive features, we studied transcriptome and proteome alterations elicited upon *IER5L* silencing. PC3 cells transduced with IER5L-targeting shRNA exhibited robust changes in the transcriptional landscape (GSE249359, Supplementary Table S2, Supplementary Fig. S7a). By comparing PC3 cells transduced with either shScramble or shIER5L lentivirus, we identified 120 differentially expressed genes (DEGs) (Fig. 5a). Of those, 63 were upregulated and 57 downregulated in *IER5L*-silenced cells. *IER5L* silencing also altered the levels of 126 proteins, 59 of which increased while 67 decreased upon silencing (Fig. 5b, Supplementary Table S3, Supplementary Fig. S7b). Of note, the combined analysis of transcriptomics and proteomics revealed 6 candidate genes that were consistently downregulated at the mRNA and protein level (Fig. 5c). The alteration in those 6 genes upon *IER5L* silencing was validated in independent sample sets (Fig. 5d, Supplementary Fig. S7c), where the different silencing efficacy of *IER5L*-targeting shRNAs was reflected in a milder effect on the expression of the genes evaluated. Moreover, the downregulation of the targets was validated using a second cell line, 22RV1 (Supplementary Fig. S7f). A decrease on *HK2*, *PTGES3* and *RCC2* was observed in this PCa cell line upon *IER5L* silencing.

Functional enrichment analysis of the molecular alterations elicited upon *IER5L* silencing in PC3 cells uncovered changes in DNA replication and monomeric G protein activity (Fig. 5e, f), which are in line with the reduction in cell growth and motility observed in our cell biology assays. Interestingly, Regulator of Chromosome Condensation 2 (RCC2) and Rho Guanine Nucleotide Exchange Factor 1 (ARHGEF1), two of the top candidate genes downregulated upon *IER5L* silencing (Fig. 5c), are regulators of the aforementioned processes [19–21]. Therefore, we studied whether the downregulation of these factors could be a contributing event to the phenotype of *IER5L* silencing. To this end, we silenced independently RCC2 and ARHGEF1 with 2 independent siRNAs and evaluated the biological consequences (Fig. 5g, h). Consistent with the consequences of *IER5L* silencing, siRNA-mediated targeting of RCC2 or ARHGEF1 reduced foci-formation but not regular two-dimensional growth (Fig. 5g, h, Supplementary Fig. S7e, f). These results suggest that the transcriptional program elicited upon IER5L targeting is a key contributing event for the tumor suppressive phenotype.

**Fig. 6:**
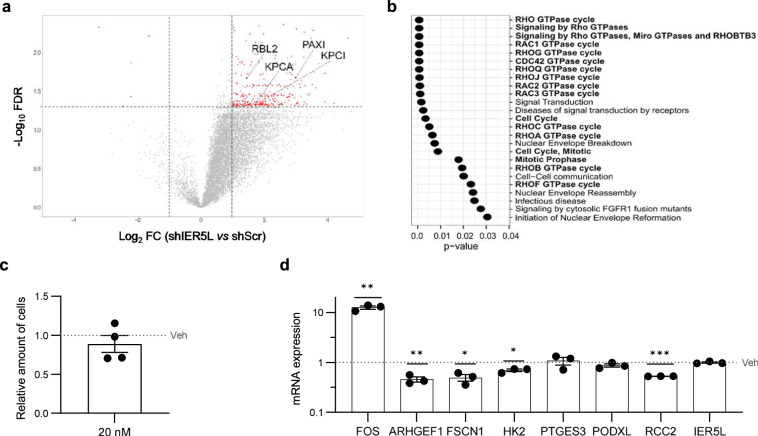
IER5L depletion elicits molecular alterations consistent with PP2A regulation. **a** Volcano plot representation of the phosphopeptides altered upon *IER5L* silencing by shScramble (shScr) or sh2 IER5L (shIER5L) transduction. The proteins inferred from the altered phosphopeptides that are reported targets of PP2A are highlighted. **b** Functional enrichment analysis of the proteins inferred from the differentially phosphorylated peptides upon *IER5L* silencing by Reactome. **c** Analysis of crystal violet staining after a 24-h treatment with 20 nM okadaic acid. The absorbance was normalized to Vehicle (Veh). A one-sample *t*-test was applied for statistical analysis (*n* = 4). **d** Analysis of the expression of the indicated genes by qRT-PCR upon a 24-h treatment with 20 nM okadaic acid. The levels are normalized to *GAPDH* and Vehicle (Veh). The dotted line represents the normalized value of the Veh data. A one-sample *t*-test was applied for statistical analysis (*n* = 3).

The information regarding the mechanism of action of the IER family genes points to the regulation of PP2A, whose function and targets are at least in part modulated by IER family members [5–8]. To study the relevance of this phosphatase in the action of IER5L, we performed two independent approaches. On one hand, we analyzed the changes in phosphopeptides elicited upon *IER5L* silencing using label-free proteomics. In line with the role of IER5L sustaining PP2A activity, we observed a robust increase in phosphopeptides upon silencing of this gene in PC3 cells (Fig. 6a, Supplementary Table S4). The inference of the proteins whose phosphorylation status changed upon *IER5L* silencing identified reported PP2A targets such as RB Transcriptional Corepressor Like 2 (RBL2) [22, 23], Protein Kinase C Iota (KPCI) [24], Protein Kinase C Alpha (KPCA) [25] and Paxilin (PAXI) [26] (Fig. 6a). Interestingly, the functional enrichment analysis of those proteins highlighted processes related to the biological and molecular consequences of *IER5L* silencing (cell cycle and monomeric G protein activity) (Fig. 6b). On the other hand, we evaluated whether pharmacological inhibition of PP2A using okadaic acid [27–30] would mimic the transcriptional alterations elicited upon *IER5L* silencing. Upregulation of Fos Proto-Oncogene (FOS) upon okadaic acid treatment [31] corroborated the activity of this compound at doses that did not elicit cytotoxicity (Fig. 6c, d). Importantly, PP2A inhibition reduced the expression of IER5L-regulated genes, including *RCC2* and *ARGHEF1*, without altering *IER5L* levels (Fig. 6d). In line with the decrease on RCC2 observed upon *IER5L* silencing on 22RV1 cells (Supplementary Fig. S7d), PP2A inhibition in this cell line recapitulated the downregulation effect on RCC2 at doses that caused no defect in cell viability (Supplementary Fig. S8). Overall, our results are consistent with biological and transcriptional effects of IER5L that are associated to the reported regulation of PP2A.

## Discussion

The identification of cellular and molecular features that can inform about the outcome of the disease entails an urgent unmet clinical need to anticipate the disease trajectory of cancer patients [2]. This notion, together with the need to decipher the molecular nodes and drivers of tumor aggressiveness is crucial to reduce the high mortality rates due to the emergence of metastasis. In this study, we identify *IER5L* as a gene that is upregulated in different cancer types, including PCa, and that IER5L upregulation contributes to the maintenance of metastatic properties of PCa tumor cells in vitro and in vivo.

IER5L is a member of the IER family that shares homology at its N-terminal domain with IER2 and IER5 [5, 32], which reportedly contribute to different aspects of cancer biology [9–13]. Unlike IER2 and IER5, the role of IER5L has been poorly studied and thus, we lack basic understanding of the possible contribution of IER5L to tumor progression and metastasis. In this regard, here we show that IER5L depletion influences the proliferation under stress, migration and invasion ability of PCa cells. *IER5L* silencing compromises tumor growth and decreases the capability of prostate cancer cells to form colonies in foci-formation assays as well as the anchorage-independent growth, while the population doubling rate in 2D unchallenged conditions remains unperturbed. This data suggests that IER5L sustains proliferative processes when cells face stressful conditions rather than in exponential growth. IER5L depletion also limits the migration and invasion ability of PCa cells, as well as their ability to disseminate and metastasize in zebrafish and murine models. The variety of the effects observed upon its silencing is consistent with the idea of IER5L being a modulator of multi-faceted cellular factors such as PP2A.

Mechanistically, our results indicate that the tumor suppressive phenotype elicited upon *IER5L* targeting is associated to changes in the transcriptional program of proliferation and monomeric G protein regulators such as RCC2 and ARHGEF1 [19–21]. In addition, *IER5L* silencing effectively increased the number of phosphorylated peptides and, in particular, peptides derived from well-known PP2A targets such as RBL2 [22, 23], KPCI [24], KPCA [25] and PAXI [26], which argue in favor of the reported evidence for IER family members, according to which IER5L acts as a positive modulator of PP2A activity. However, we cannot rule out the existence of other molecular mechanisms of action of IER5L beyond the regulation of the phosphatase.

PP2A is a versatile and important phosphatase involved in cell division and its inhibition has a profound impact on cellular proliferation [33]. Yet, the effect of IER5L depletion on cell proliferation is limited to specific experimental conditions, suggesting that the functional interaction between PP2A and IER5L is complex. Although we did not observe gene expression alteration of *IER2* and IER5 upon *IER5L* silencing (data not shown), we cannot rule out that other family members could compensate for the loss of IER5L in specific cellular settings through the regulation of PP2A [5].

The results obtained in this study reveal that IER5L expression is altered in PCa and other cancers, and that its levels contribute to cancer-related biological processes. IER5L depletion elicits transcriptional changes with biological consequences consistent with the reported reduction in PP2A activity. Further studies around the mechanism of regulation and function of IER5L could provide relevant information to deconstruct the molecular bases of cancer pathogenesis and progression.

## Materials and methods

### Cell culture and treatments

PC3 and DU145 cells were purchased at Leibniz-Institut DSMZ-Deutsche Sammlung con Mikroorganismen und Zelkulturen GmbH with the corresponding certificate of authenticity. 22RV1 were purchased from American Type Culture Collection, who provided authentication certificate. TRAMP-C1 cell line was kindly provided by Dr. Marco Piva. PC3, DU145, HEK293FT and TRAMP-C1 cells were cultured in Dulbecco’s modified eagle medium (DMEM) (Gibco™; 41966-029). 22RV1 cells were maintained in Roswell park memorial institute (RPMI 1640; 11875-093) medium. Both culture mediums were supplemented with 10% of inactivated fetal bovine serum (FBS) (Gibco™; 10270-106) and 1% of penicillin/streptomycin (Gibco™; 15140-122). In the case of TRAMP-C1 cells, 10% of Antibiotic-Antimycotic (Gibco™; 15240062) was used instead of (Gibco™; 15140-122). Cell lines were tested for mycoplasma contamination routinely using MycoAlert detection Kit (Lonza; LT07-318).

### siRNA transfections

1 × 10^6^ PC3 cells were reverse-transfected with Lipofectamine 2000 (ThermoFisher; 11668027) and 20 nM of the indicated small interference RNA (siRNA)(Sigma-Aldrich) following manufacturer’s guidelines. A pool of 4 sequences was used to target IER5L. A pool of 4 non-targeting siRNAs was used in parallel as a control. Individual siRNAs were used for ARHGEF1 and RCC2 experiments. All the sequences are detailed in Supplementary Table S5.

### Lentiviral production and cell line generation

HEK293FT cells were used for lentiviral production. Lentiviral vectors expressing short hairpins (shRNAs) against human and murine Scramble and IER5L were purchased from Sigma-Aldrich. Cells were transfected with lentiviral vectors following standard procedures. Puromycin (2 μg/ml; Sigma-Aldrich; P8833) was used for selection. The shRNA sequences are detailed in Supplementary Table S6.

### Real-time quantitative RT-PCR (qRT-PCR)

Total RNA was isolated using Maxwell RSC simplyRNA Cells Kit (Promega; AS1390). Complementary DNA was produced from 1 μg of RNA using Maxima™ H Minus cDNA Synthesis Master Mix (Invitrogen; M1682,). Amplifications were run in a Viia7, QS5 or QS6 Real-Time PCR Systems (Applied Biosystems) using the following Taqman probes from Life Technologies: IER5L/Ier5l (Hs04186822_s1/ Mm07299543_s1), ARHGEF1 (Hs00180327_m1), FSCN1 (Hs00602051_mH), HK2 (Hs00606086_m1), PODXL (Hs01574644_m1), PTGES3 (Hs04187819_g1) and RCC2 (Hs00603046_m1). Universal Probe Library (Roche, Basel, Switzerland) primers and probes employed (Roche; ThermoFisher) for FOS detection: Forward (ctaccactcacccgcagact), reverse (aggtccgtgcagaagtcct) and probe number 67. The expression of individual genes was calculated and normalized to the expression of GAPDH/Gapdh (Hs02758991_g1/ Mm99999915_g1) or β-Actin (Mm00607939_s1) as indicated.

### Cell culture assays

#### Proliferation assays

Five thousand cells were seeded in 12-well plates on triplicate. Cells were fixed with 10% formalin (Avantor) after 0, 3 and 6 days and stained with crystal violet (0.1% in 20% methanol; Sigma-Aldrich) for 30 min.

#### Foci-formation assays

Five hundred cells/well were seeded in a 6-well plate on triplicate. Cells were fixed with 10% formalin (Avantor) and stained with 0.1% crystal violet (Sigma-Aldrich) in 20% methanol after 10 days. The number of visible foci were manually counted.

#### Anchorage-independent growth

Anchorage-independent growth ability was measured by means of soft-agar colony formation assay [34]. Briefly, a six-well tissue culture dish was coated with the bottom agar layer (complete DMEM containing 0.6% agar) and stored at 4 °C for ≥30 min to let the agar solidify. Cells (TRAMP-C1: 375 cells/well and PC3: 2500 cells/well) were resuspended in a 0.3% low-melting-point agar (Agarose LM, Conda) and plated on top of the bottom agar. The plates were incubated (37 °C and 5% CO_2_) for 3 weeks for growth of colonies. Colonies were imaged using an Olympus IX-83 inverted microscope operated by CellSens software and the number of visible colonies was counted using Fiji software.

#### Wound healing assays

Wound healing assays were performed by seeding 2 × 10^5^ cells in 6-well plates at high confluency. A 20 µL pipet tip was used to wound the cell monolayer. Images were acquired at 0, 24, and 32- h using an Olympus IX-83 inverted microscope operated by CellSens software. Cell migration rate was measured by calculating the linear growth of wound closure and dividing the slope by 2 times the length of the initial area of the scratch. Experiments were performed on triplicate. Fiji software was used to quantify the wounded area.

#### Invasive growth assays

Seven hundred cell drops of 25 µL were prepared in DMEM with 6% methylcellulose (Sigma-Aldrich; M0387). Drops were incubated at 37 °C and 5% CO_2_ for 48 h. Once formed, spheroids were collected, resuspended in collagen I solution (Advanced BioMatrix PureCol; 5005), and added to 12-well plates. Five experimental replicates were used for each condition. Four hours later, media was added, and pictures were taken at 0 and 72- h using an Olympus IX-83 inverted microscope operated by CellSens software. Invasion growth was calculated as area difference on day 3 versus day 0 using FiJi software.

### Animals

#### Zebrafish

Zebrafish experiments were carried out as previously described [18]. In brief, 230 PC3-GL cells expressing shScramble or sh2 IER5L were resuspended in PBS at 0.5 × 10^8^ cells /ml concentration. 48 zebrafish embryos (Casper strain [35]) at 2 days post fecundation were anesthetized with tricaine, mounted in agarose and microinjected with 4.6 nl cancer cells (approximately 230 cells/embryo) into the common cardinal vein. After microinjection, the xenografted embryos were placed in E3-medium supplemented with 0.2 mM N-Phenylthiourea (Sigma-Aldrich; P7629) and penicillin-streptomycin (Sigma-Aldrich; P4333) and incubated at 33 °C. After overnight incubation, the embryos were anesthetized and GFP-positive embryos with successful intravascular injection were placed into 96-well plates for imaging (1 embryo/well). Imaging was done using Nikon Eclipse Ti2-E widefield microscope using 2× Plan-Apochromat objective (NA0.06), fluorescence (ex475/28 nm LED, Chroma 84000v2 DAPI/FITC/TRITC/Cy5 Quad filter) and brightfield illumination. The number of disseminated cells was counted manually using Fiji.

#### Mice

All mouse experiments were carried out following the ethical guidelines established by the Biosafety and Animal Welfare Committee at CIC bioGUNE (Spanish acronym for center for cooperative research in Biosciences). The procedures employed were carried out following the recommendations from the Association for Assessment and Accreditation of Laboratory Animal Care (AAALAC). For orthotopic assays, 5 × 10^5^ PC3-GL cells expressing shScramble or sh2 IER5L cells were injected into the ventral prostate lobes of 10 Nu/Nu immunodeficient males of 6–12 weeks of age. One mouse died during the procedure. The Tumor growth and dissemination of the other 9 Nu/Nu mice was followed by measuring bioluminescence for up to 20 days with IVIS technology (PerkinElmer). Intra-orbital injections of 50 µL luciferase at 15 mg/mL were used during the follow-up. Luciferase signal and tumor weight were measured at the time of sacrifice on day 20 after injection. Cell dissemination was analyzed in mice organs ex vivo. Bioluminescence signal above 10^7^ was considered as positive for metastatic lesions.

### RNA-Seq analysis

Samples for RNA-Seq and Proteomic analysis were seeded in parallel. Four 100-mm plates of shScramble or sh2 IER5L cells were seeded at 80% confluency for each experiment. RNA was extracted using Maxwell RSC simplyRNA Cells Kit (Promega; AS1390) and, after RNA QC, Total RNA libraries were prepared at the Genomics platform of CIC bioGUNE using *TruSeq Stranded Total RNA with Ribo-Zero Globin* kit (Illumina Inc., Cat.# 20020612) and *TruSeq RNA CD Index Plate* (Illumina Inc., Cat.# 20019792) following “TruSeq Stranded Total RNA Sample Prep-guide (Part # 15031048 Rev. E)”. Libraries were sequenced on an Illumina NovaSeq 6000 instrument to generate at least 40 million of paired-end 100 bp reads.

Reads were aligned to the human reference genome (hg38) using STAR (version_2.7.5c) in two-pass mode following STAR best practices and recommendations [36] The quality of the data was evaluated using STAR (version 2.7.5c) [36] and samtools (version 1.15) [37]. PCR duplicates were removed from aligned bam files using samtools (version 1.15) [37]. Read counts were extracted from the aligned bam files using subread’s FeatureCounts (version 2.0.3) [38]. Normalization of read counts for analysis was done according to EdgeR recommendations using the Ratio of the Variance method which accounts for inter-sample variance and the differential expression analysis of the normalized read counts between the sample groups was performed following best practices and recommendations of EdgeR [39, 40] and Limma [41] on R environment (version 3.6.0). All the code used for data analysis is available upon request. An FDR < 0.05 and a log2 fold change (FC) of 0.5 were used to address significant differences within the groups and to select the DEGs.

### Proteomic and phosphoproteomic analysis

#### Sample preparation

For proteomic analysis, 20 µg of protein/sample were extracted in a buffer containing 7 M urea, 2 M Thiourea 4% 3-((3-cholamidopropyl) dimethylammonio)-1-propanesulfonate (CHAPS) and 5 mM Dithiothreitol (DTT). Protein extracts were digested following the filter-aided FASP protocol described by Wisniewski et al. with minor modifications [42]. Trypsin was added to a trypsin:protein ratio of 1:20, and the mixture was incubated overnight at 37°C, dried out in a RVC2 25 speed vac concentrator (Christ), and resuspended in 0.1% Formic acid (FA). Peptides were desalted and resuspended in 0.1% FA using C18 stage tips (Millipore).

For phosphoproteomic analysis, 3 million cells were seeded and processed following the FASP protocol described above. Phosphopeptides were purified using the High-Select™ TiO_2_ Phosphopeptide Enrichment Kit (ThermoFisher) following the manufacturer’s instructions. Samples were directly loaded onto the mass spectrometer.

#### Mass spectrometry analysis

Samples were analyzed in a timsTOF Pro with PASEF (Bruker Daltonics) coupled online to an Evosep ONE (Evosep) liquid chromatograph. Total protein samples were loaded in 0.1% FA, whereas phosphoproteomic samples were directly loaded in a solution containing approximately 5% TFA. Both sample types were resolved using the 30 samples-per-day protocol (15 cm column and 44 min gradient runs).

#### Protein and phosphoprotein identification and quantification

Protein identification and quantification was carried out using PEAKS X software (Bioinformatics solutions). Searches were carried out against a database consisting of *Homo sapiens* entries (Uniprot/Swissprot), with precursor and fragment tolerances of 20 ppm and 0.05 Da. Carbamidomethylation of cysteines was considered as fixed modification, and oxidation of methionine as variable. Phosphorylation of serine, threonine and tyrosine were considered as variable modifications in the phosphoproteomics dataset.

Protein or phosphopeptide abundances inferred from PEAKS were loaded onto Perseus platform [43]. These values were log2 transformed and only those proteins or phosphopeptides present in at least 70% of the samples of any of the groups under analysis were considered for further analyses. Missing values were imputed using values coming from the 10% less abundant proteins or phosphopeptides in each sample.

#### Data analysis and functional enrichment analysis

A *t*-test (*p* < 0.05) for proteins and a *t*-test (*q* value < 0.05) for phosphoproteins was used to address significant differences within each sample group under analysis. A log2 FC of 0.5 and a log 2 FC of 1 were used to identify the proteins and phosphoproteins altered by IER5L depletion, respectively.

Database for Annotation, Visualization and Integrated Discovery (DAVID) [44] web interface was used for functional enrichment analysis. The whole list of proteins identified in our proteomic analysis was used as background for KEGG analysis. The Reactoma analysis was performed using Homo sapiens as background in DAVID.

### Statistical analysis

No statistical method was used to predetermine the sample size. The experiments were not randomized. The investigators were not blinded to allocation during experiments and outcome assessment. None of the samples/animals was excluded from the analysis. Data analyzed by parametric tests are represented by the mean ± SEM. of pooled experiments unless otherwise stated. Values of *n* represent the number of independent experiments performed or the number of individual mice or patient specimens. For each independent in vitro experiment, at least three technical replicates were used, and a minimum number of three experiments were performed to ensure adequate statistical power. In the in vitro experiments, normal distribution was assumed, and one sample *t*-test was applied for one-component comparisons with control and Student’s *t*-test for two-component comparisons. For in vivo experiments, a non-parametric Mann-Whitney *U*-test was used. Two-tailed statistical analysis was applied for experimental design without predicted result, and one-tail for validation or hypothesis-driven experiments. The confidence level used for all the statistical analyses was 0.95 (alpha value = 0.05). *p-*value: *p < 0.05, **p < 0.01, ***p < 0.001.

## Supplementary information

Supplementary Figures and legends

Supplementary Tables

## Data Availability

The authors declare that data supporting the findings of this study are available within the paper and its supplementary files.

## References

[1] Ferlay J, Soerjomataram I, Dikshit R, Eser S, Mathers C, Rebelo M, et al. Cancer incidence and mortality worldwide: sources, methods and major patterns in GLOBOCAN 2012. Int J Cancer. 2015;136:E359–86. 25220842 10.1002/ijc.29210

[2] Sidransky D. Emerging molecular markers of cancer. Nat Rev Cancer. 2002;2:210–9. 11990857 10.1038/nrc755

[3] Rebello RJ, Oing C, Knudsen KE, Loeb S, Johnson DC, Reiter RE, et al. Prostate cancer. Nat Rev Dis Prim. 2021;7:9. 33542230 10.1038/s41572-020-00243-0

[4] Healy S, Khan P, Davie JR. Immediate early response genes and cell transformation. Pharmacol Ther. 2013;137:64–77. 22983151 10.1016/j.pharmthera.2012.09.001

[5] Ueda T, Kohama Y, Sakurai H. IER family proteins are regulators of protein phosphatase PP2A and modulate the phosphorylation status of CDC25A. Cell Signal. 2019;55:81–9. 30599213 10.1016/j.cellsig.2018.12.012

[6] Doi K, Takeuchi H, Sakurai H. PP2A-B55 and its adapter proteins IER2 and IER5 regulate the activity of RB family proteins and the expression of cell cycle-related genes. FEBS J. 2023;290:745–62. 36047562 10.1111/febs.16612

[7] Ishikawa Y, Kawabata S, Sakurai H. HSF1 transcriptional activity is modulated by IER5 and PP2A/B55. FEBS Lett. 2015;589:1150–5. 25816751 10.1016/j.febslet.2015.03.019

[8] Kawabata S, Ishita Y, Ishikawa Y, Sakurai H. Immediate-early response 5 (IER5) interacts with protein phosphatase 2A and regulates the phosphorylation of ribosomal protein S6 kinase and heat shock factor 1. FEBS Lett. 2015;589:3679–85. 26496226 10.1016/j.febslet.2015.10.013

[9] Asano Y, Kawase T, Okabe A, Tsutsumi S, Ichikawa H, Tatebe S, et al. IER5 generates a novel hypo-phosphorylated active form of HSF1 and contributes to tumorigenesis. Sci Rep. 2016;6:19174. 26754925 10.1038/srep19174 PMC4709660

[10] Neeb A, Wallbaum S, Novac N, Dukovic-Schulze S, Scholl I, Schreiber C, et al. The immediate early gene Ier2 promotes tumor cell motility and metastasis, and predicts poor survival of colorectal cancer patients. Oncogene. 2012;31:3796–806. 22120713 10.1038/onc.2011.535

[11] Wu W, Zhang X, Liao Y, Zhang W, Cheng H, Deng Z, et al. miR-30c negatively regulates the migration and invasion by targeting the immediate early response protein 2 in SMMC-7721 and HepG2 cells. Am J Cancer Res. 2015;5:1435–46. 26101708 PMC4473321

[12] Wu W, Zhang X, Lv H, Liao Y, Zhang W, Cheng H, et al. Identification of immediate early response protein 2 as a regulator of angiogenesis through the modulation of endothelial cell motility and adhesion. Int J Mol Med. 2015;36:1104–10. 26260137 10.3892/ijmm.2015.2310

[13] Xu Z, Zhu L, Wu W, Liao Y, Zhang W, Deng Z, et al. Immediate early response protein 2 regulates hepatocellular carcinoma cell adhesion and motility via integrin beta1-mediated signaling pathway. Oncol Rep. 2017;37:259–72. 27840969 10.3892/or.2016.5215

[14] Cortazar AR, Torrano V, Martin-Martin N, Caro-Maldonado A, Camacho L, Hermanova I, et al. CANCERTOOL: a visualization and representation interface to exploit cancer datasets. Cancer Res. 2018;78:6320–8. 30232219 10.1158/0008-5472.CAN-18-1669

[15] Wang N, Tan X, Cao S, Liu M. Predictive value of immediate early response 5 like (IER5L) in the prognosis and immune checkpoint blockade therapy of non-small cell lung cancer patients. Pathol Res Pract. 2024;256:155270. 38552564 10.1016/j.prp.2024.155270

[16] Chen X, He YQ, Miao TW, Yin J, Liu J, Zeng HP, et al. IER5L is a prognostic biomarker in pan-cancer analysis and correlates with immune infiltration and immune molecules in non-small cell lung cancer. Int J Gen Med. 2023;16:5889–908. 38106972 10.2147/IJGM.S439190 PMC10725786

[17] Li T, Fan J, Wang B, Traugh N, Chen Q, Liu JS, et al. TIMER: a web server for comprehensive analysis of tumor-infiltrating immune cells. Cancer Res. 2017;77:e108–e10. 29092952 10.1158/0008-5472.CAN-17-0307 PMC6042652

[18] Paatero I, Alve S, Gramolelli S, Ivaska J, Ojala PM. Zebrafish embryo xenograft and metastasis assay. Bio Protoc. 2018;8:e3027. 34395813 10.21769/BioProtoc.3027 PMC8328589

[19] Yenjerla M, Panopoulos A, Reynaud C, Fotedar R, Margolis RL. TD-60 is required for interphase cell cycle progression. Cell Cycle. 2013;12:837–41. 23388455 10.4161/cc.23821 PMC3610731

[20] Bouafia A, Lofek S, Bruneau J, Chentout L, Lamrini H, Trinquand A, et al. Loss of ARHGEF1 causes a human primary antibody deficiency. J Clin Invest. 2019;129:1047–60. 30521495 10.1172/JCI120572 PMC6391114

[21] Mollinari C, Reynaud C, Martineau-Thuillier S, Monier S, Kieffer S, Garin J, et al. The mammalian passenger protein TD-60 is an RCC1 family member with an essential role in prometaphase to metaphase progression. Dev Cell. 2003;5:295–307. 12919680 10.1016/s1534-5807(03)00205-3

[22] Garriga J, Jayaraman AL, Limon A, Jayadeva G, Sotillo E, Truongcao M, et al. A dynamic equilibrium between CDKs and PP2A modulates phosphorylation of pRB, p107 and p130. Cell Cycle. 2004;3:1320–30. 15467457 10.4161/cc.3.10.1183

[23] Jayadeva G, Kurimchak A, Garriga J, Sotillo E, Davis AJ, Haines DS, et al. B55alpha PP2A holoenzymes modulate the phosphorylation status of the retinoblastoma-related protein p107 and its activation. J Biol Chem. 2010;285:29863–73. 20663872 10.1074/jbc.M110.162354 PMC2943288

[24] Ugi S, Imamura T, Maegawa H, Egawa K, Yoshizaki T, Shi K, et al. Protein phosphatase 2A negatively regulates insulin’s metabolic signaling pathway by inhibiting Akt (protein kinase B) activity in 3T3-L1 adipocytes. Mol Cell Biol. 2004;24:8778–89. 15367694 10.1128/MCB.24.19.8778-8789.2004 PMC516764

[25] Ricciarelli R, Azzi A. Regulation of recombinant PKC alpha activity by protein phosphatase 1 and protein phosphatase 2A. Arch Biochem Biophys. 1998;355:197–200. 9675027 10.1006/abbi.1998.0732

[26] Ito A, Kataoka TR, Watanabe M, Nishiyama K, Mazaki Y, Sabe H, et al. A truncated isoform of the PP2A B56 subunit promotes cell motility through paxillin phosphorylation. EMBO J. 2000;19:562–71. 10675325 10.1093/emboj/19.4.562 PMC305594

[27] Arias C, Sharma N, Davies P, Shafit-Zagardo B. Okadaic acid induces early changes in microtubule-associated protein 2 and tau phosphorylation prior to neurodegeneration in cultured cortical neurons. J Neurochem. 1993;61:673–82. 8336148 10.1111/j.1471-4159.1993.tb02172.x

[28] Bialojan C, Takai A. Inhibitory effect of a marine-sponge toxin, okadaic acid, on protein phosphatases. Specificity and kinetics. Biochem J. 1988;256:283–90. 2851982 10.1042/bj2560283 PMC1135400

[29] Ishihara H, Martin BL, Brautigan DL, Karaki H, Ozaki H, Kato Y, et al. Calyculin A and okadaic acid: inhibitors of protein phosphatase activity. Biochem Biophys Res Commun. 1989;159:871–7. 2539153 10.1016/0006-291x(89)92189-x

[30] Takai A, Bialojan C, Troschka M, Ruegg JC. Smooth muscle myosin phosphatase inhibition and force enhancement by black sponge toxin. FEBS Lett. 1987;217:81–4. 3036577 10.1016/0014-5793(87)81247-4

[31] Schonthal A, Tsukitani Y, Feramisco JR. Transcriptional and post-transcriptional regulation of c-fos expression by the tumor promoter okadaic acid. Oncogene. 1991;6:423–30. 1901402

[32] Williams M, Lyu MS, Yang YL, Lin EP, Dunbrack R, Birren B, et al. Ier5, a novel member of the slow-kinetics immediate-early genes. Genomics. 1999;55:327–34. 10049588 10.1006/geno.1998.5679

[33] Wlodarchak N, Xing Y. PP2A as a master regulator of the cell cycle. Crit Rev Biochem Mol Biol. 2016;51:162–84. 26906453 10.3109/10409238.2016.1143913 PMC4905575

[34] Hermanova I, Zuniga-Garcia P, Caro-Maldonado A, Fernandez-Ruiz S, Salvador F, Martin-Martin N. Genetic manipulation of LKB1 elicits lethal metastatic prostate cancer. J Exp Med. 2020;217:e20191787. 32219437 10.1084/jem.20191787 PMC7971141

[35] White RM, Sessa A, Burke C, Bowman T, LeBlanc J, Ceol C, et al. Transparent adult zebrafish as a tool for in vivo transplantation analysis. Cell Stem Cell. 2008;2:183–9. 18371439 10.1016/j.stem.2007.11.002 PMC2292119

[36] Dobin A, Davis CA, Schlesinger F, Drenkow J, Zaleski C, Jha S, et al. STAR: ultrafast universal RNA-seq aligner. Bioinformatics. 2013;29:15–21. 23104886 10.1093/bioinformatics/bts635 PMC3530905

[37] Danecek P, Bonfield JK, Liddle J, Marshall J, Ohan V, Pollard MO. et al. Twelve years of SAMtools and BCFtools. Gigascience. 2021;10:1–4. 10.1093/gigascience/giab008 PMC7931819 33590861

[38] Liao Y, Smyth GK, Shi W. featureCounts: an efficient general purpose program for assigning sequence reads to genomic features. Bioinformatics. 2014;30:923–30. 24227677 10.1093/bioinformatics/btt656

[39] Chen Y, Lun AT, Smyth GK. From reads to genes to pathways: differential expression analysis of RNA-Seq experiments using Rsubread and the edgeR quasi-likelihood pipeline. F1000Res. 2016;5:1438. 27508061 10.12688/f1000research.8987.1 PMC4934518

[40] Robinson MD, McCarthy DJ, Smyth GK. edgeR: a Bioconductor package for differential expression analysis of digital gene expression data. Bioinformatics. 2010;26:139–40. 19910308 10.1093/bioinformatics/btp616 PMC2796818

[41] Ritchie ME, Phipson B, Wu D, Hu Y, Law CW, Shi W, et al. limma powers differential expression analyses for RNA-sequencing and microarray studies. Nucleic Acids Res. 2015;43:e47. 25605792 10.1093/nar/gkv007 PMC4402510

[42] Wisniewski JR, Zougman A, Nagaraj N, Mann M. Universal sample preparation method for proteome analysis. Nat Methods. 2009;6:359–62. 19377485 10.1038/nmeth.1322

[43] Tyanova S, Temu T, Sinitcyn P, Carlson A, Hein MY, Geiger T, et al. The Perseus computational platform for comprehensive analysis of (prote)omics data. Nat Methods. 2016;13:731–40. 27348712 10.1038/nmeth.3901

[44] Sherman BT, Hao M, Qiu J, Jiao X, Baseler MW, Lane HC, et al. DAVID: a web server for functional enrichment analysis and functional annotation of gene lists (2021 update). Nucleic Acids Res. 2022;50:W216–W21. 35325185 10.1093/nar/gkac194 PMC9252805

