# Correction: The PP2A regulator IER5L supports prostate cancer progression

**DOI:** 10.1038/s41419-025-07802-x

**Published:** 2025-07-22

**Authors:** Jana R. Crespo, Natalia Martín-Martín, Saioa Garcia-Longarte, Jon Corres-Mendizabal, Onintza Carlevaris, Ianire Astobiza, Amaia Zabala-Letona, Marc Guiu, Mikel Azkargorta, Monika Gonzalez-Lopez, Nuria Macías-Cámara, Phuong Doan, Félix Elortza, Isabel Mendizabal, Jukka Westermarck, Roger R. Gomis, Amaia Ercilla, Arkaitz Carracedo

**Affiliations:** 1 Center for Cooperative Research in Biosciences (CIC bioGUNE), Basque Research and Technology Alliance (BRTA), Bizkaia Technology Park, Derio, Spain; 2 Traslational prostate cancer Research lab, CIC bioGUNE-Basurto, Biobizkaia Health Research Institute, Bizkaia, Spain; 3 Centro de Investigación Biomédica En Red de Cáncer (CIBERONC), Madrid, Spain; 4 Cancer Science Program, Institute for Research in Biomedicine (IRB Barcelona), The Barcelona Institute of Science and Technology, Barcelona, Spain; 5 Proteomics Platform, CIC bioGUNE, Basque Research and Technology Alliance (BRTA), Gipuzkoa, Spain; 6 CIBERehd, Bizkaia Science and Technology Park, Derio, Spain; 7 Turku Bioscience Centre, University of Turku and Åbo Akademi University, Turku, Finland; 8 IKERBASQUE, Basque Foundation for Science, Bilbao, Spain; 9 Institute of Biomedicine and InFLAMES Research Flagship, University of Turku, Turku, Finland; 10 School of Medicine, Universitat de Barcelona, Barcelona, Spain; 11 ICREA, Institució Catalana de Recerca i Estudis Avançats, Barcelona, Spain; 12 Biochemistry and Molecular Biology Department, University of the Basque Country (UPV/EHU), Bilbao, Spain

**Keywords:** Cell signalling, Metastasis

Correction to: *Cell Death & Disease* 10.1038/s41419-024-06907-z, published online 18 July 2024

The author Jukka Westermarck was initially written as Jukka Westermack.

The original article has been corrected.

