# Supplementary material for: The PP2A regulator IER5L supports prostate cancer progression
Source: Cell Death Dis. 2024 Jul 18;15(7):514. doi: 10.1038/s41419-024-06907-z (PMC11258296; doi:10.1038/s41419-024-06907-z)
Supplement: Supplementary file 1 — Supplementary Figures and legends [file 41419_2024_6907_MOESM1_ESM.pdf]

Supplementary Figures

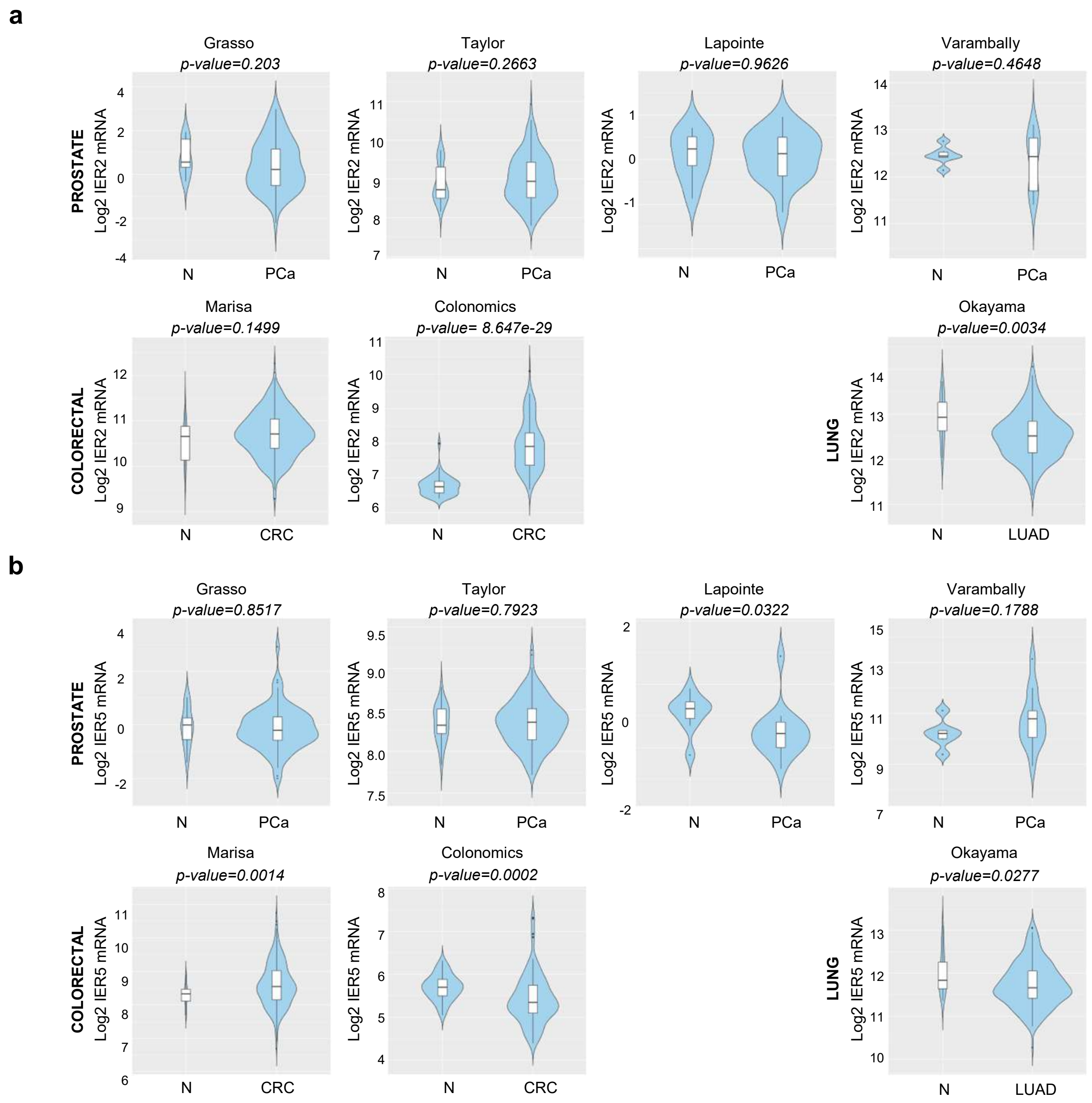

Supplementary Figure S1. ***Related to Fig.1*. IER2 and IER5 levels in non-tumoral and tumoral specimens.** Violin plots depicting the Log2 expression of (**a**) *IER2* and (**b**) *IER5* in non-tumoral (N), prostate cancer (PCa), lung adenocarcinoma (LUAD) and colorectal (CRC) specimens in the indicated dataset. *p*-value derives from a Student's *t*-test analysis between the indicated groups.

a

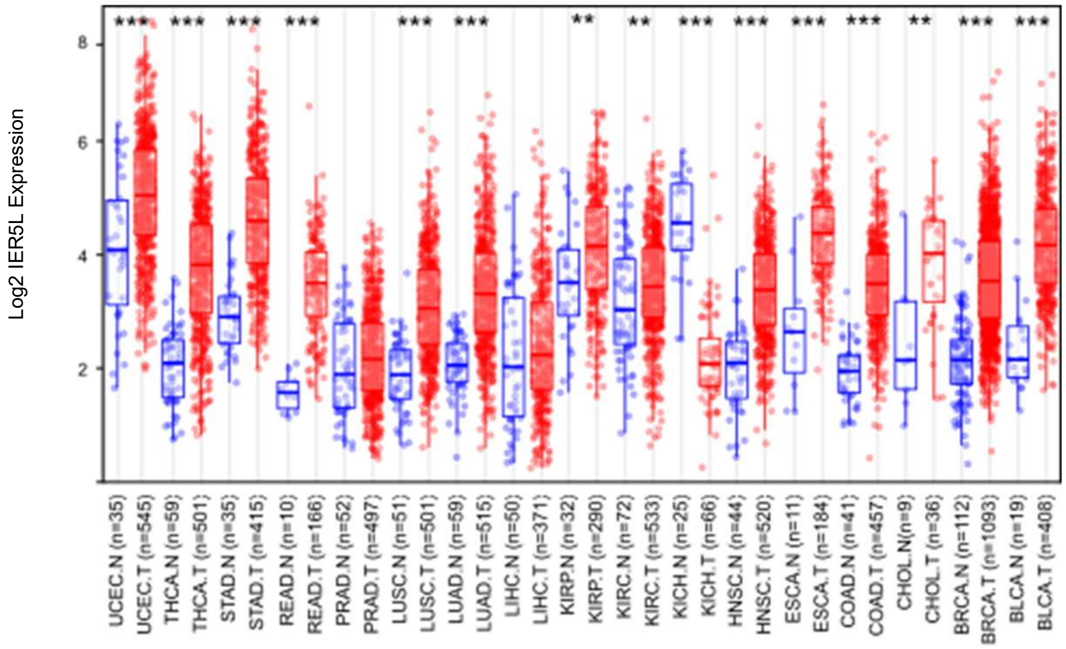

b

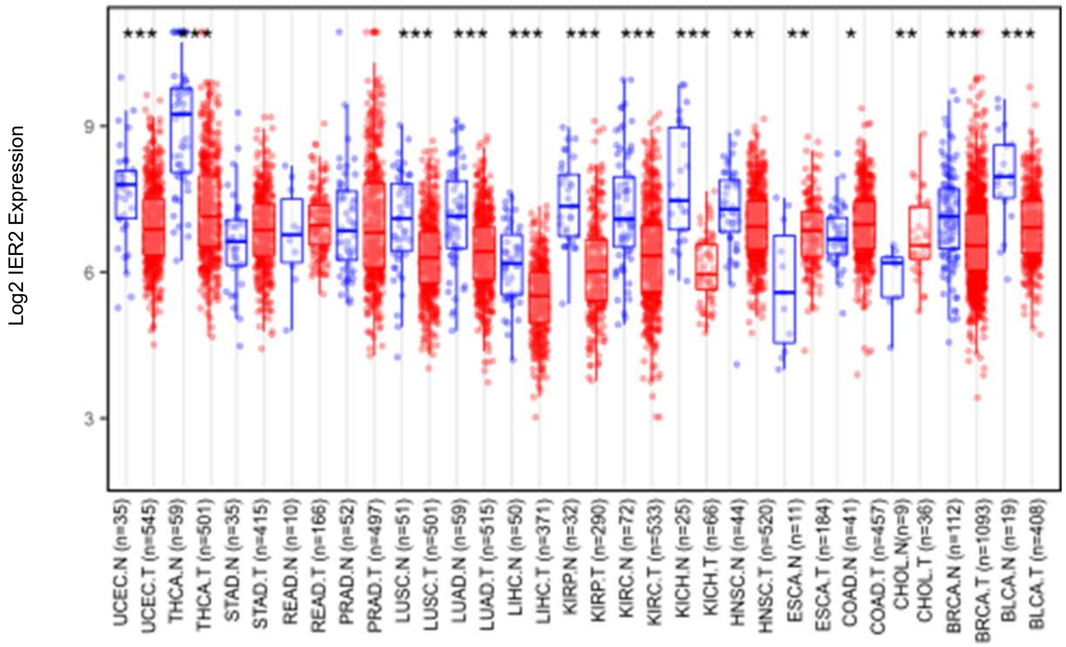

c

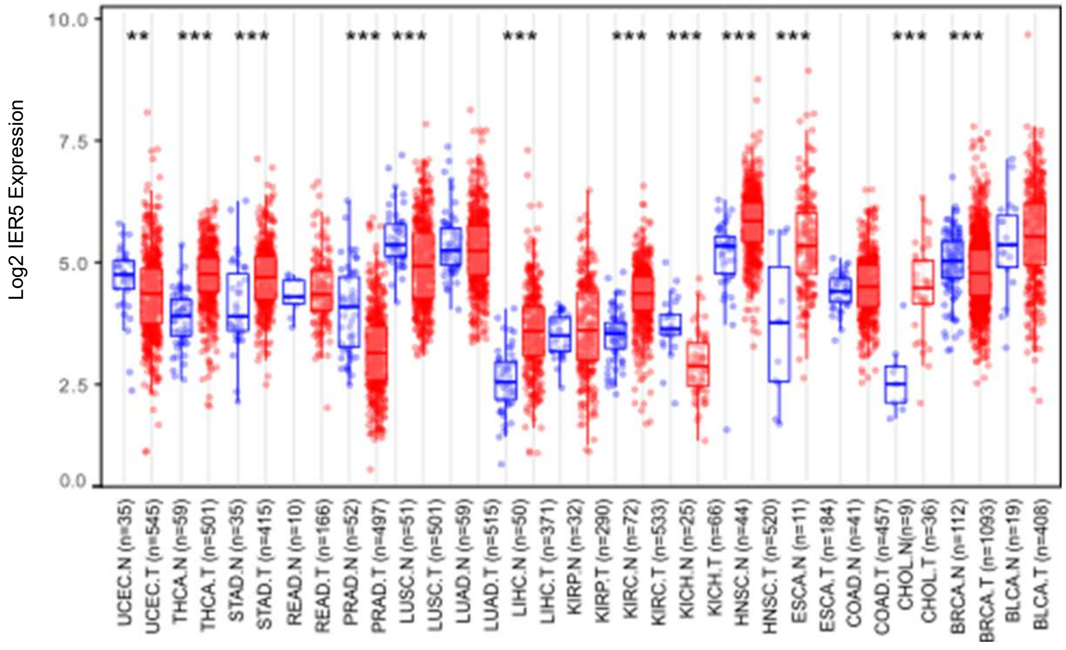

Supplementary Figure S2. *Related to Fig.1.* Analysis of IER5L, IER2 and IER5 expression in cancer.
TIMER 2.0 analysis of *IER5L* (a), *IER2* (b) and *IER5* (c) levels in non-tumoral (N) and tumoral (T) specimens of the indicated cancer type (TCGA cohort). A Wilcoxon test was applied for statistical analysis.

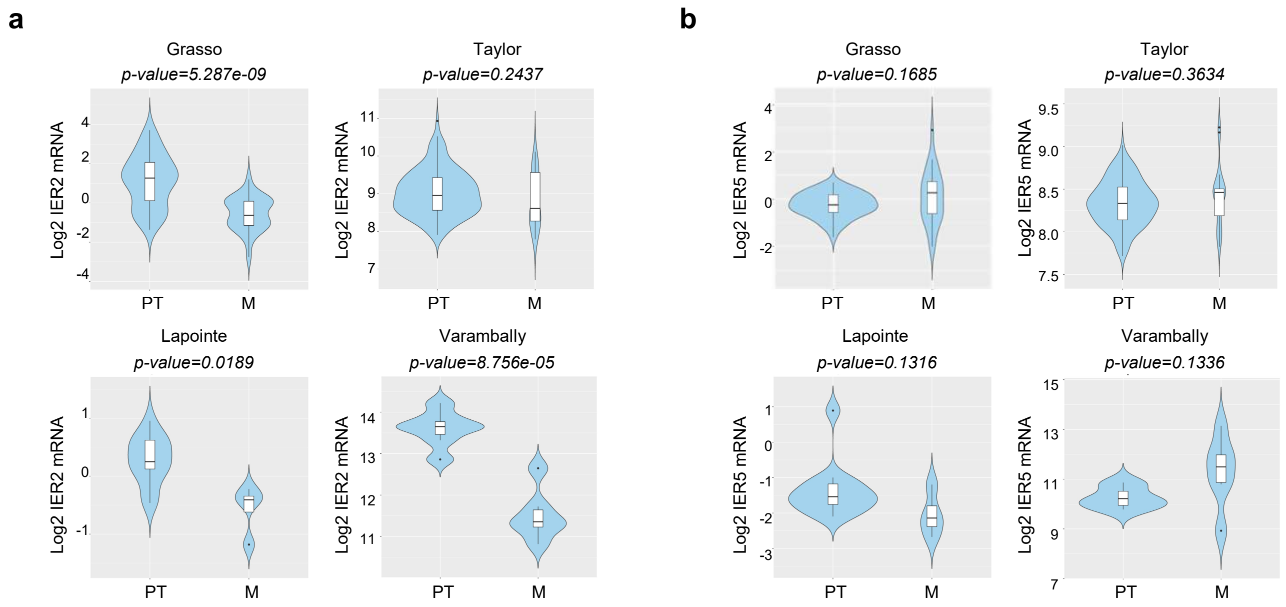

Supplementary Figure S3. ***Related to Fig.1.*** **Analysis of IER2 and IER5 expression in metastatic prostate cancer.** Violin plots showing the Log2 expression of *IER2* (**a**) and *IER5* (**b**) in primary tumor (PT) and metastatic (M) PCa specimens. *p*-value derives from a Student's *t*-test analysis between the indicated groups.

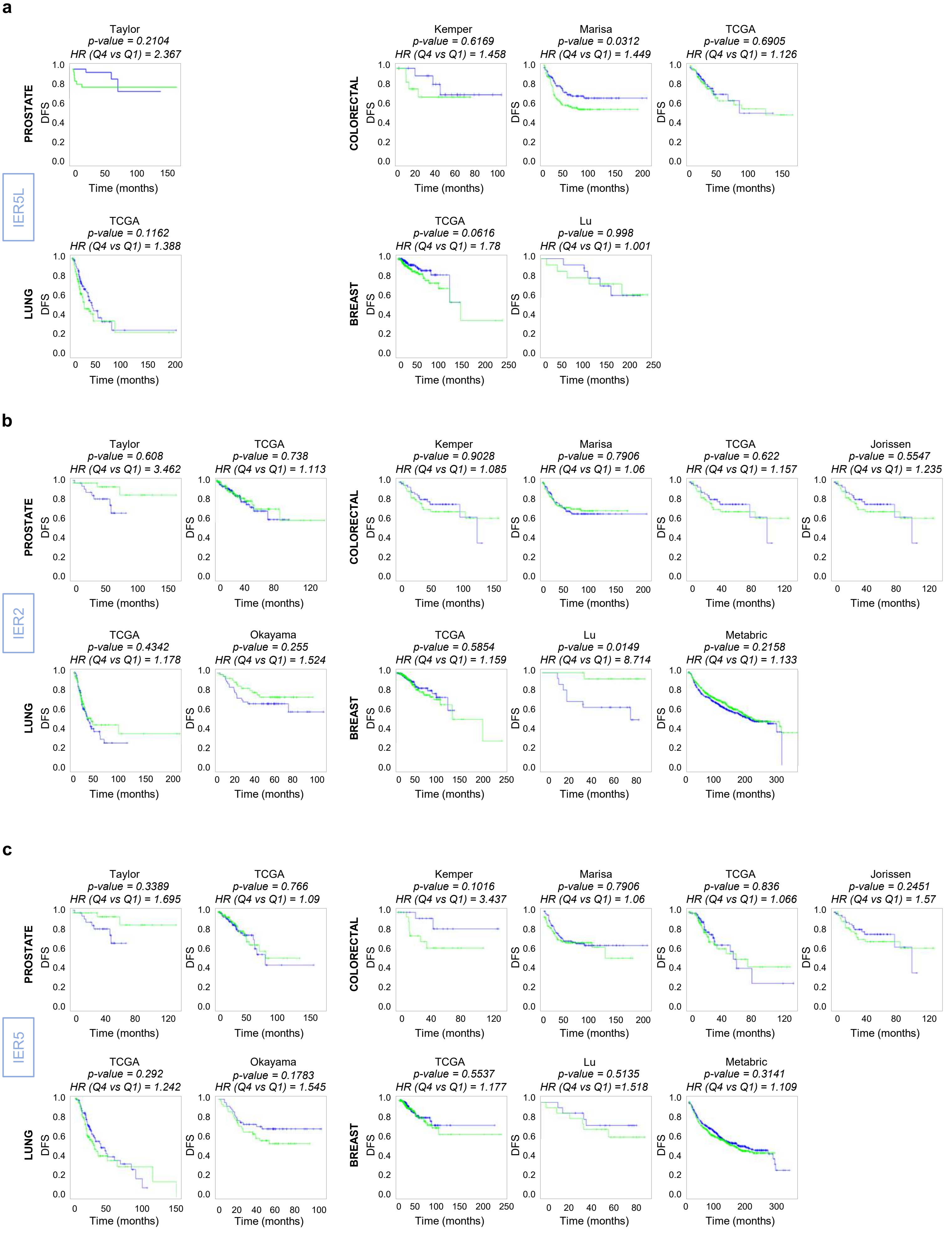

Supplementary Figure S4. ***Related to Fig.1.*** **Evaluation of the prognostic potential of the IER family genes.**
Kaplan-Meyer curves showing the association of *IER5L* (**a**), *IER2* (**b**) and *IER5* (**c**) mRNA expression to disease-free survival (DFS) in the indicated datasets. Quartile 1 (blue) and quartile 4 (green) are represented. A log-rank test *p*-value and the hazard ratio (HR) between two groups calculated by a Cox proportional hazard regression model are provided above each graph.

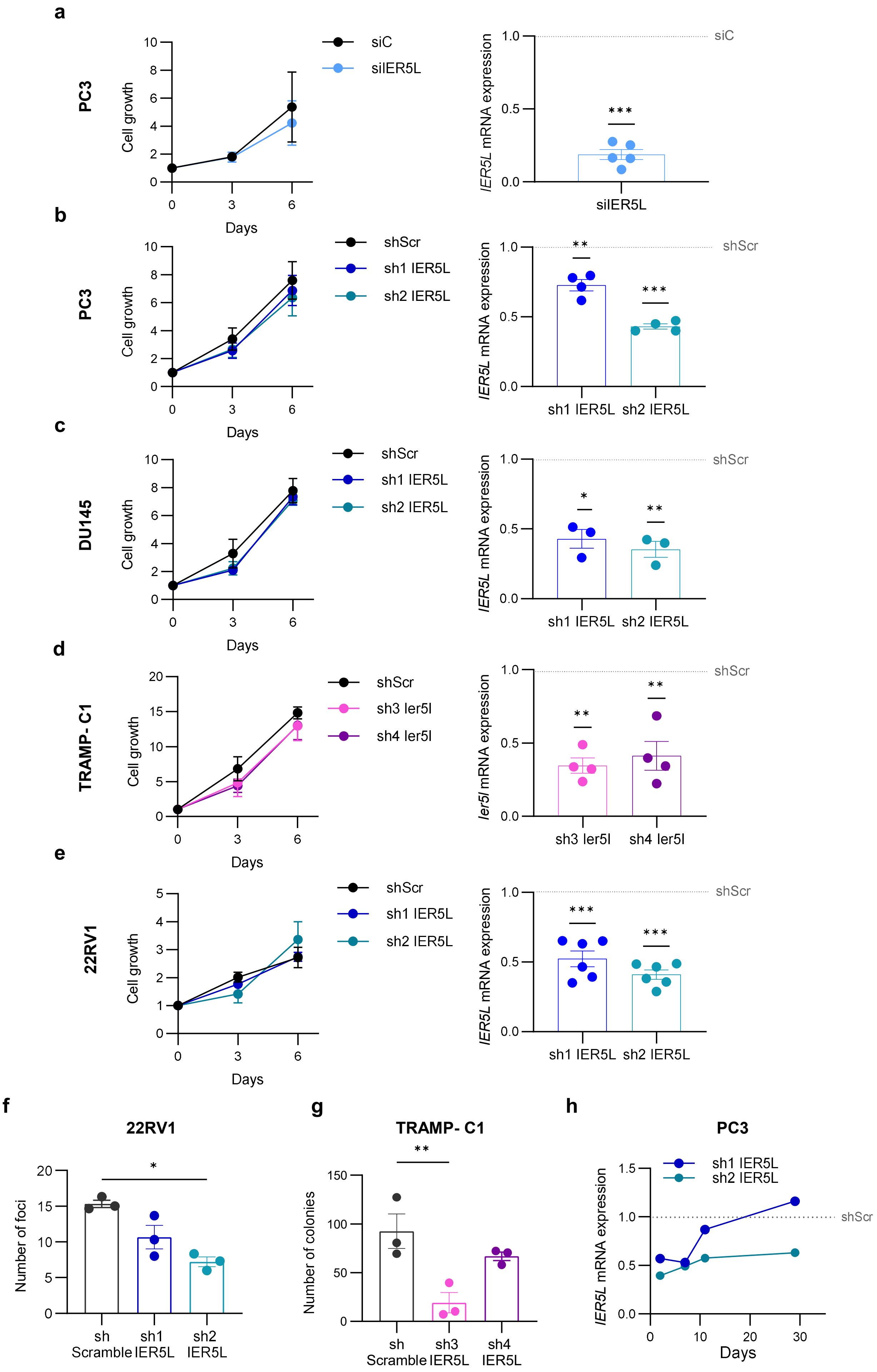

Supplementary Figure S5. ***Related to Fig.2.*** **IER5L depletion does not impair the proliferation of prostate cancer cells.**
a-e Analysis of cell proliferation upon *IER5L* silencing by the indicated siRNAs or shRNAs. 2 days after siRNA transfection were defined as the initial timepoint for the assay. Left panels: The cell number at each time point relative to time 0 are represented. A multiple paired *t*-test was applied for statistical analysis. Right panels: Analysis of *IER5L* mRNA expression by qRT-PCR. The levels of *IER5L* are normalized to *GAPDH* and data show the expression levels relative to non-target siRNA (**a** siC) or shScramble (**b** shScr). The dotted line represents the normalized value of the siC or shScr data. A one sample *t*-test was applied for statistical analysis. PC3 (**a** *n*=5; **b** *n*=4), DU145 (*n*=3), TRAMP-C1 (*n*=4) and 22RV1 (*n*=6).

f Analysis of foci formation upon IER5L depletion with the indicated shRNAs. The number of foci is shown. A paired *t*-test was applied for statistical analysis (*n*=3).

g Analysis of anchorage-independent growth ability of TRAMP-C1 cells transduced with the indicated shRNAs. The number of colonies is shown. A paired *t*-test was applied for statistical analysis (*n*=3).

h Analysis of *IER5L* mRNA levels by qRT-PCR overtime. PC3 cells were transduced with the indicated shRNAs and followed up over several passages. The data of a representative experiment is shown. The dotted line represents the normalized value of the shScr cell data.

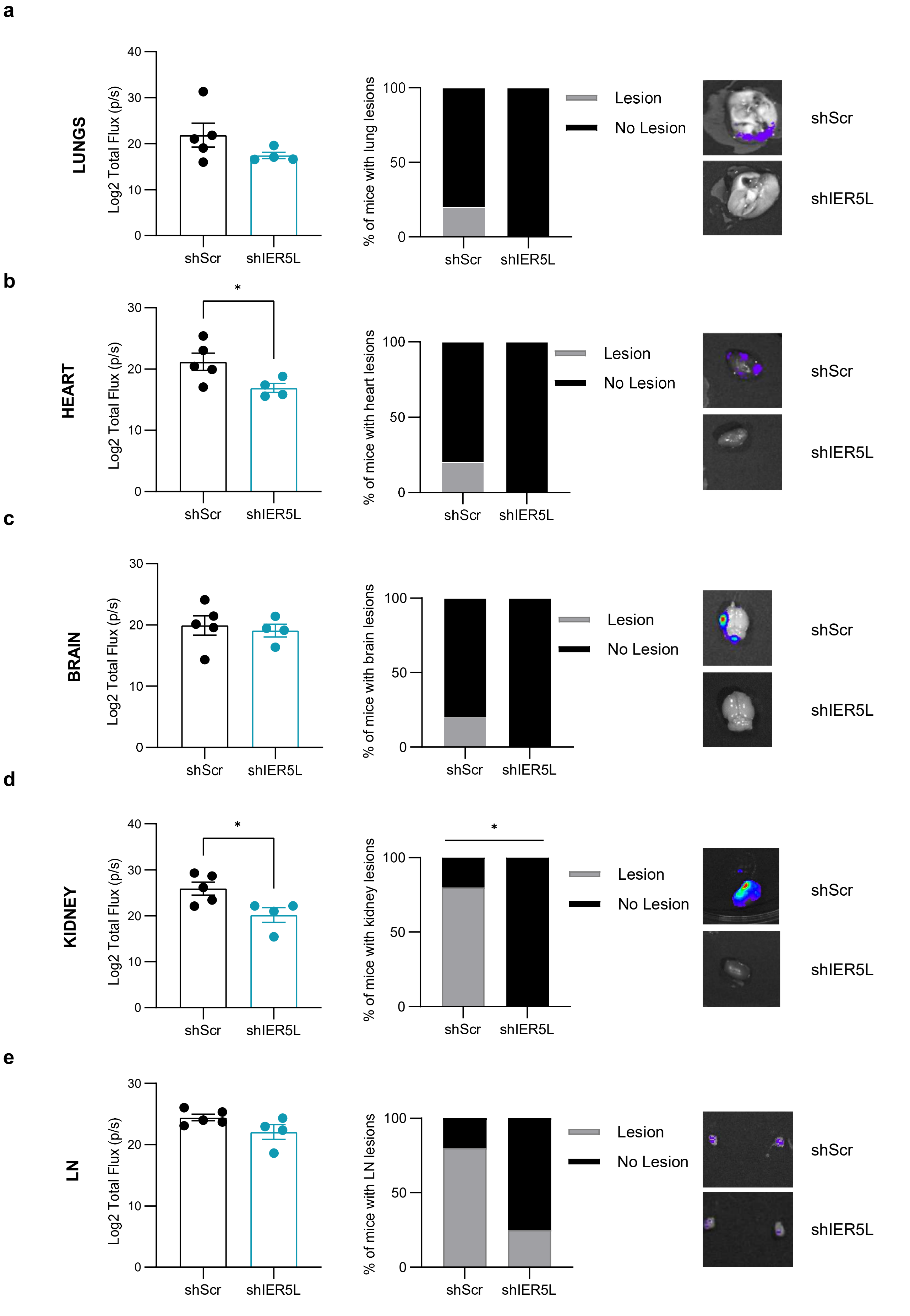

Supplementary Figure S6. ***Related to Fig.4.*** **Metastatic dissemination of prostate cancer cells.**
Ex vivo IVIS signal quantification of lungs (**a**), heart (**b**), brain (**c**), kidney (**d**) and lymph nodes (**e** LN) (left panels). Contingency analysis of metastatic lesions at those sites (middle panels) and a representative image (right panels) are shown. A one-tailed Mann-Whitney test and Fisher exact *t*-test were used, respectively.

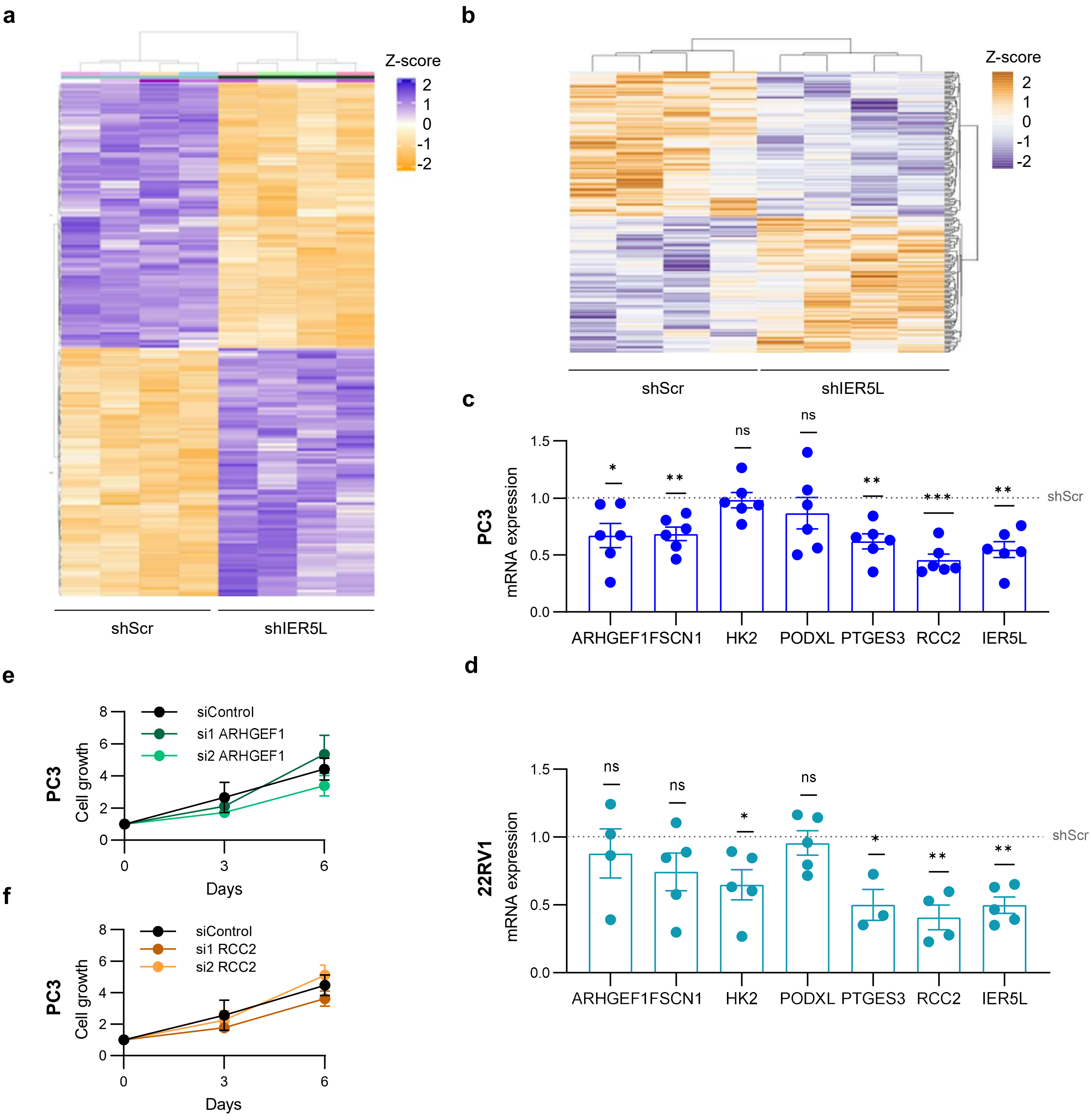

Supplementary Figure S7. ***Related to Fig.5.*** **Transcriptomics and proteomics landscape of *IER5L*-silenced cells.**
a and b PC3 cells were transduced with shScramble (shScr) or sh2 IER5L (shIER5L). The Z-scored heatmaps representing the effect of *IER5L* silencing on mRNA (**a**) and protein (**b**) levels are represented. Only significant changes are represented ($p<0.05$). In (**a**) only the Top200 significant ones are shown.
c Analysis of the expression of the indicated genes by qRT-PCR upon *IER5L* depletion by sh1 IER5L transduction in PC3 cells. The mRNA levels are normalized to *GAPDH* and shScr. The dotted line represents the normalized value of the shScr data. A one sample *t*-test was applied for statistical analysis (n=6).
d Analysis of the expression of the indicated genes by qRT-PCR upon *IER5L* depletion by sh2 IER5L transduction in 22RV1 cells. The mRNA levels are normalized to *GAPDH* and shScr. The dotted line represents the normalized value of the shScr data. A one sample *t*-test was applied for statistical analysis (n=3-5).
e and f Analysis of cell proliferation upon *ARHEGF1* (**e**) or *RCC2* (**f**) silencing in PC3 cells. The cell number at each time point relative to time 0 is represented. A multiple paired *t*-test was applied for statistical analysis (n=4).

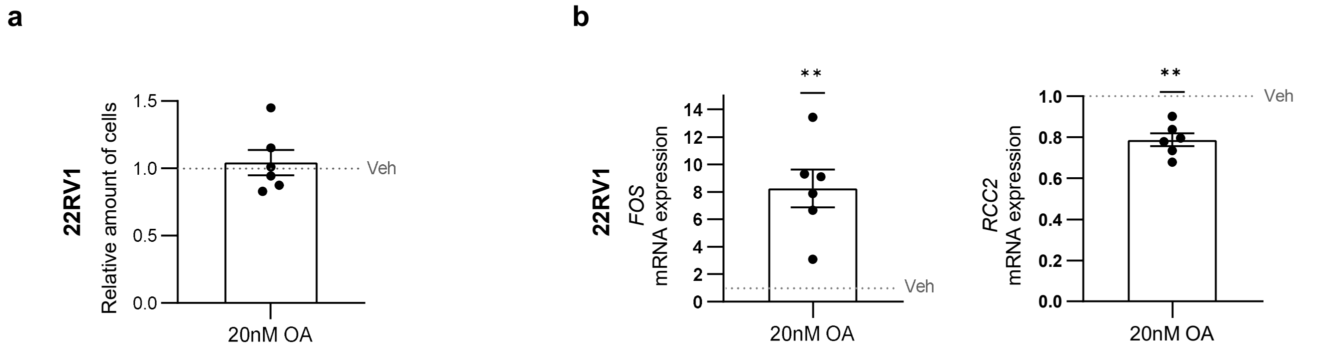

Supplementary Figure S8. ***Related to Fig.6.*** **Effect of PP2A inhibition on IER5L target genes.**

a) Analysis of crystal violet staining after a 24 hour treatment with 20 nM okadaic acid. The absorbance was normalized to Vehicle (Veh). A one sample *t*-test was applied for statistical analysis (*n*=6).

b) Analysis of the expression of the indicated genes by qRT-PCR upon a 24 hour treatment with 20 nM okadaic acid. The levels are normalized to *GAPDH* and Veh. The dotted line represents the normalized value of the Veh data. A one sample *t*-test was applied for statistical analysis (*n*=6).
